# Earthquake-Related Injuries in the Pediatric Population: A Systematic Review

**DOI:** 10.1371/currents.dis.6d3efba2712560727c0a551f4febac16

**Published:** 2013-11-27

**Authors:** Gabrielle A. Jacquet, Bhakti Hansoti, Alexander Vu, Jamil D. Bayram

**Affiliations:** Boston University School of Medicine, Boston, Massachusetts, USA; Johns Hopkins University, Baltimore, Maryland, USA; Johns Hopkins University, Baltimore, Maryland, USA; Johns Hopkins University, Baltimore, Maryland, USA; Johns Hopkins Office of Critical Event Preparedness and Response, Baltimore, Maryland, USA; Center for Refugee and Disaster Response, Bloomberg School of Public Health, Johns Hopkins University, Baltimore, Maryland, USA

## Abstract

Background: Children are a special population, particularly susceptible to injury. Registries for various injury types in the pediatric population are important, not only for epidemiological purposes but also for their implications on intervention programs. Although injury registries already exist, there is no uniform injury classification system for traumatic mass casualty events such as earthquakes. Objective: To systematically review peer-reviewed literature on the patterns of earthquake-related injuries in the pediatric population. Methods: On May 14, 2012, the authors performed a systematic review of literature from 1950 to 2012 indexed in Pubmed, EMBASE, Scopus, Web of Science, and Cochrane Library. Articles written in English, providing a quantitative description of pediatric injuries were included. Articles focusing on other types of disasters, geological, surgical, conceptual, psychological, indirect injuries, injury complications such as wound infections and acute kidney injury, case reports, reviews, and non-English articles were excluded. Results: A total of 2037 articles were retrieved, of which only 10 contained quantitative earthquake-related pediatric injury data. All studies were retrospective, had different age categorization, and reported injuries heterogeneously. Only 2 studies reported patterns of injury for all pediatric patients, including patients admitted and discharged. Seven articles described injuries by anatomic location, 5 articles described injuries by type, and 2 articles described injuries using both systems. Conclusions: Differences in age categorization of pediatric patients, and in the injury classification system make quantifying the burden of earthquake-related injuries in the pediatric population difficult. A uniform age categorization and injury classification system are paramount for drawing broader conclusions, enhancing disaster preparation for future disasters, and decreasing morbidity and mortality.

## Introduction

Traumatic injuries are the leading cause of death among persons 10-19 years of age worldwide^1^, and among persons 1-44 years of age in the United States.^2^ Injury registries are necessary, not only for epidemiological purposes but also for their implications on designing intervention programs and disaster preparedness. Several injury registries have been developed in the past, such as the Web-based Injury Statistics Query and Reporting System (WISQARS)^2^ by the Centers for Disease Control and Prevention (CDC), and the American College of Surgeons National Trauma Data Bank (NTDB)^3^. However there is currently no uniform international database for injuries sustained in natural disasters such as earthquakes, neither in adult nor pediatric populations. In order to provide robust disaster preparedness and an optimized response, this information must be available in an organized repository. The objective of this article is to systematically review the peer-reviewed literature regarding the pattern of earthquake-related injuries in the pediatric population, and to use these findings to provide recommendations for improving reporting and classification of pediatric injuries in disasters.

## Methods

*Study Design: *The authors performed a systematic review by searching literature from 1950 to 2012 indexed in Pubmed, Embase, Scopus, Web of Science and the Cochrane Library. All searches were performed on May 14, 2012. A comprehensive search strategy was developed and translated into each databases' syntax. The search was limited to medical related subject areas in Scopus and Web of Science to limit off-topic literature retrieved due to the wider scope of those databases. The search included an earthquake concept and a trauma concept. Terms were searched as controlled vocabulary in applicable databases (Pubmed, Embase, Cochrane) and as keywords in all databases (**Appendix 1**). All terms were searched as exact phrases in all databases. The search method algorithm is detailed in **Figure 1**.

**Methods Algorithm:**
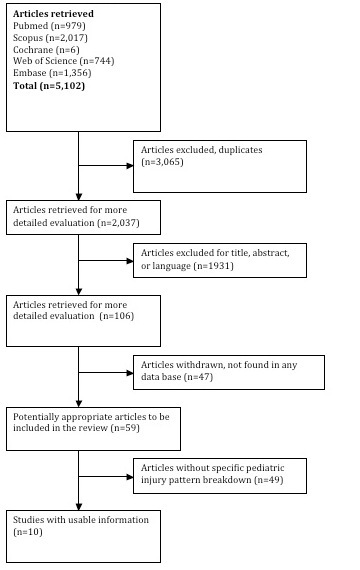

Results from the five databases were combined and duplicates were excluded, yielding a total of 2,037 articles. Title screening was performed to identify articles that were unrelated to natural disasters or human populations. Each title was screened by three reviewers and was retained if any of the reviewers established that inclusion criteria were met. During review of article titles, consensus was met when two out of three agreed on the relevance of the titles to advance in the systematic review. *Inclusion Criteria: *Articles written in English providing a quantitative description of the types of direct physical injuries sustained immediately in the aftermath of earthquakes, and specifying the ages of injured patient*s. Exclusion Criteria: *Articles about other types of disasters, geological, surgical, and conceptual articles, articles describing psychological impact, indirect injuries, injury complications such as wound infections and acute kidney injury, case reports, reviews, and/or articles in languages other than English. A total of 106 articles underwent full review by all three authors; all three authors agreed on the final 10 articles included in this review.****

## Results

A total of 2,037 articles were retrieved, of which only 10 (0.49%) contained quantitative data on earthquake-related pediatric injuries and could be used in the final analysis.^4^^,^ 5 ^,^ 6 ^,^ 7 ^,^ 8 ^,^ 9 ^,^ 10 ^,^ 11 ^,^ 12 ^,^ 13 These articles and their characteristics are outlined in **Table 1**. All 10 studies were retrospective. Studies had different pediatric age group upper limits ranging from 14 to 18 years. The average number of pediatric patients in the 10 studies was 124 (range 33-254). Each study reported pediatric injuries using heterogeneous categories and classifications; using injury type (e.g. fracture) or location (e.g. head, upper limb, trunk), or a combination of both. Only five studies focused solely on pediatric patients, two of which reported patterns of injury for all pediatric patients, whether admitted to the hospital or discharged after initial treatment. Only one article described the injuries by anatomic location, and one described injuries by type; the remaining eight articles described injuries using a variety of combinations of both systems.

**Table 1: Article Characteristics:** 

Author	Title	Year Article Pub- lished	Site of Earth- quake	Year of Earth- quake	Age Group of Study Popu- lation	Number of Injured Pediatric Patients Studied	Additional Study Population Characteristics	Age of Pediatric Popu- lation
Gueri M	The Popayan earthquake: A preliminary report on its effect on health	1984	Columbia	1983	all	41	Analysis of admitted patients only	<15 yrs
Reyes Ortiz M	Brief description of the effects on health of the earthquake of 3rd March 1985 -- Chile	1986	Chile	1985	all	254		<15 yrs
Sanchez-Carillo CI	Morbidity Following Mexico City's 1985 Earthquakes	1989	Mexico	1985	all	128		<15 yrs
Iskit SH	Analysis of 33 Pediatric Trauma Victims in the 1999 Marmara, Turkey Earthquake	2001	Turkey	1999	pediatric	33	Analysis of pts transferred to referral hospital	14 days- 16 yrs
Sarisozen B	Extremity Injuries in Children resulting from the 1999 Marmara earthquake: an epidemiologic study	2003	Marmara	1999	all	51	Admitted patients only	<16 yrs
Sabzehchian M	Pediatric Trauma at Tertiary-Level Hospitals in the Aftermath of the Bam, Iran Earthquake	2006	Iran	2003	pediatric	119	Analysis of pts admitted to 3 referral hospitals	<16 yrs
Bai X	Retrospective analysis: the earthquake-injured patients in Barakott of Pakistan	2009	Pakistan	2005	all	151		9 mos- 16 yrs
Xiang B	Triage of pediatric injuries after the 2008 Wen-Chuan earthquake in China	2009	China	2008	pediatric	119	Admitted patients only	pre- school, school
Farfel A	Haiti earthquake 2010: a field hospital pediatric perspective	2011	Haiti	2010	pediatric	155		0-18 yrs
Zhao J	Sichaun Earthquake and Emergency relief care for children	2011	Sichuan	2008	pediatric	192		<18 yrs

Fractures were the most commonly identified type of injury (four of the seven articles) with reported percentages ranging from 18.1% to 55.2% (pooled percentage 30.6%).^9^^,^ 10 ^,^ 12 ^,^ 13 Soft tissue injuries were the second most common type of injury, ranging from 17.6% to 70.2%.^4^^,^ 5 ^,^ 8 Pooled percentages could not be calculated for this type of injury because universal nomenclature was not employed when reporting. Crush injuries were frequently cited, ranging from 6.3% to 18.7% (pooled percentage 20.4%). Special mention was made to the secondary consequences of renal failure and the need for dialysis. **Table 2** illustrates the specific classification of Injury by article, where possible, injury percentages are presented alongside the raw data.

Many of the articles that reported injury by location focused only on orthopedic injuries, with fracture of extremities accounting for 17.1% to 60.8% (pooled percentage 36.8%).^4^^,^ 8 ^,^ 9 ^,^ 11 ^,^ 12 ^,^ 13 A single article identified the head as the most common location for trauma.^6^ The percentage of head injuries ranged from 3.2% to 61% (pooled percentage 18.4%). The incidence of reported spinal trauma ranged between 4.9% and 31.1% (pooled percentage 6.5%).^7^^,^ 9 ^,^ 1

**Table 2: Injury Classification Systems:** 

Author	Year Article Published	Number of Injured Pediatric Patients Studied	Injuries Classified by Type	#	% of Injured Patients	Injuries Classified by Location	#	% of Injured Patients
Gueri M	1984	41	multiple trauma	5	12.2%	head injury	25	61.0%
			fracture	10	24.4%	lower limb	4	9.8%
						spinal column	2	4.9%
						upper limb	3	7.3%
						other	1	2.4%
			traumatism (not specified)	1	2.4%	intraabdominal/ thoracic	1	2.4%
Reyes Ortiz M	1986	254	fracture	46	18.1%	skull bones and face	1	0.4%
						neck and trunk	5	2.0%
						upper extremity	12	4.7%
						lower extremity	28	11.0%
			dislocation	0	0.0%	intracranial injury without fracture	27	10.6%
			sprains and tears	7	2.8%	internal injury to chest/abdomen/pelvis	2	0.8%
			wound	104	40.9%	head, neck, trunk	55	21.7%
						upper extremity	14	5.5%
						lower extremity	35	13.8%
			superficial injury	0	0.0%			
			contusion without alteration of skin	46	18.1%			
			bruises	4	1.6%			
			injury to nerves and spinal column	0	0.0%			
			complication of unspecified injury	2	0.8%			
			other	14	5.5%			
Sanchez-Carillo CI	1989	128	multiple traumas	21	16.4%			
			simple fractures	19	14.8%			
			compound fractures	2	1.6%			
			simple contusions	37	28.9%			
			crushing	0	0.0%			
			wounds with contusions	18	14.1%			
			other	13	10.2%			
Iskit SH	2001	33	crush injuries	15		CNS	8	24.2%
			soft tissue	19		vertebral column	2	6.1%
			peripheral nerve palsy	3		thoracic compression	1	3.0%
						retroperitoneal hematoma	2	6.1%
			fracture	8	24.2%	extremity	6	18.2%
						pelvis	2	6.1%
Sarisozen B	2003	51				extremity and spine	31	60.8%
						chest	1	2.0%
						abdomen	3	5.9%
						head	3	5.9%
						other	5	9.8%
						unknown	8	15.7%
Sabzehchian M	2006	119	joint injury	60	50.4%	upper/lower limb	10/50	8.4%/42.0%
			laceration	61	51.3%	upper/lower limb	5/56	4.2%/47.1%
			fracture	63	52.9%	upper/lower limb	11/52	9.2%/43.7%
			ecchymosis	40	33.6%	upper/lower limb	9/31	7.6%/26.1%
			hematoma	21	17.6%	upper/lower limb	2/19	1.7%/16.0%
			deep wound	23	19.3%	upper/lower limb	1/22	1.7%/18.5%
			vascular	13	10.9%	upper/lower limb	0/13	0.0%/10.9%
						chest and abdomen	17	14.3%
						head and spinal cord	37	31.1%
Bai X	2009	151	open soft tissue injury	106	70.2%	upper extremity wound	27	17.9%
			open fracture	6	4.0%	lower extremity wound	37	24.5%
			closed fracture	20	13.2%	head wound	34	22.5%
			pain only	19	12.6%	trunk wound	2	1.3%
						multple sites wound	12	7.9%
Xiang B	2009	119	fractures	104		upper limb	26	
						lower limb	60	
						pelvis	12	
						skull	2	
						thoracic spine	4	
			nerve injury	7				
			limb compartment syndrome	17				
			dislocation	2				
			liver fracture	5				
			soft tissue injury	4				
			hemopneumo- thorax	4				
Farfel A	2011	155	fractures	48	31.0%	head injuries	5	3.2%
			open wounds	52	33.5%			
			crush injuries	29	18.7%			
			superficial injuries	29	18.7%			
			contusion	8	5.2%			
			dislocations	5	3.2%			
			other	6	3.9%			
Zhao J	2011	192	simple, open	127	66.1%	head	23	12.0%
			simple, closed	41	21.4%	face and neck	6	3.1%
			combined open and closed	35	18.2%	chest	18	9.4%
			crush injury	12	6.3%	abdomen	6	3.1%
			fracture	106	55.2%	pelvis	13	6.8%
			soft tissue	73	38.0%	spine	17	8.9%
						limb	106	55.2%
						body surface	67	34.9%

## Discussion

The pediatric patient is likely to present with a unique array of injury patterns, secondary to differences in physiology and anatomy. By better understanding the specific injuries this population may face, healthcare providers may more adequately prepare for the needs of this vulnerable population in post-disaster settings. Therefore, trauma registries in any population, especially vulnerable subpopulations such pediatrics, are important to capture data for research, measure trauma system outcomes, and support quality improvement through assessment of the appropriateness and effectiveness of the trauma system.^14^ A trauma registry can be defined as “a disease specific collection composed of a file of uniform data elements that describe the injury event, demographics, prehospital information, diagnosis, care, outcomes, and costs of treatment for injured patients”.^15^ In most cases it is computerized, permitting ease of analysis and tracking of quality improvement data elements. It is this ease of analysis and ability to track specific data (such as complications or process-of-care measures), as well as ability to adjust for severity of injury, that distinguish trauma registries from general medical records systems.^16^

Injury Patterns in Pediatric Survivors of Earthquake

Overall our systematic review of injury patterns in the pediatric population demonstrated a high incidence of fracture-related injuries (30.6%) and wounds. Our findings that extremities were the most common site of injury (36.8%) was also reported by Bartels, et al.^17^ Head injury and spinal injury were reported in 18.4%^6^ and 6.5%^11^ of patients, respectively. It is important to note that head injury reporting varied significantly depending on the author’s classification (with or without fracture, and with or without intracranial hemorrhage).

Our review also revealed that crush injuries are consistently reported (in 4 out of 10 articles). Crush injury and crush syndrome are common earthquake injury patterns. Crush injury is defined as compression of extremities and body parts that causes muscle swelling or neurologic disturbances in the affected parts of the body. Typically affected body parts include lower extremities (74%) and upper extremities (10%).^18^ These two locations were the most commonly reported injury sites in this review: 34.2% and 12.3%, respectively.^19^

Challenges in the Evaluation of Pediatric Injury Patterns 

Our systematic review on earthquake-related pediatric injuries highlights major challenges regarding pediatric injury reporting in disaster settings. These challenges can be regarded as both limitations and urgent needs for consensus and future prospective research. The first challenge is related to the upper age limit of a "pediatric" patient in reporting injuries, as the definition of what constitutes a “child” varied significantly among these studies between 14 and 18 years of age. Consequently, that finding posed a major difficulty when compiling data, even for comparable injuries. The American College of Surgeons National Trauma Data Bank uses < age 20 years as their upper limit, however the facilities from which they receive data use anywhere from age 11 years to age 21 years, as the upper limit.^20^ The ACS also further classifies pediatric trauma information into the following age groups: < 1 year, 1-4 years, 5-9 years, 10-14, 14-19,^20^ which none of the ten articles in our systematic review reported.

The second challenge relates to the substantial heterogeneity in classifying pediatric injuries. Our final 10 articles with related earthquake-related pediatric injuries had widely different methods for how data was collected, categorized and reported. As a result, the information was very difficult to interpret, which makes injury-specific disaster planning difficult. One way to potentially circumvent this challenge would be to adopt international classification standards such as International Classification of Disease (ICD)^21^ codes, the World Health Organization (WHO) International Classification of External Causes of Injuries (ICECI)^ 24^ as well as Abbreviated Injury Scale (AIS)^22^ , and Body Region (Head, Lower Extremity, Thorax, Abdomen, Upper Extremity, Spine, External/Other, Face, Neck) notation used by the National Trauma Data Bank in the United States^3^ . International agreement on any injury classification system is an involved process, but could be undertaken and implemented by credible entities such as the WHO.

The third challenge is the technical reporting of numerical results. In five of the ten studies, it was difficult to ascertain the denominator to calculate percentages of injuries, when only raw injury counts were provided.^5^^,^ 8 ^,^ 10 ^,^ 11 ^,^ 12 For example, in the article by Xiang et al., where 119 injured pediatric patients were studied, the authors reported 17 limb compartment syndromes and 60 lower limb injuries.^12^ It was unclear from their data if these injuries are reported on different patients or if single patients accounted for more than one injury. In other words, we could not simply ascertain if a patient with bilateral lower limb injuries was counted once or twice. These numerical reporting discrepancies among various studies can have a huge epidemiologic impact.

The fourth challenge is the striking paucity of reporting pediatric-specific data in traumatic injuries. It is postulated that pediatric injuries are more likely to be underreported, due to the fact that children have a higher mortality rate, may have survived with fewer injuries or were incapable of reaching the hospital due to familial constraints.^5^ From the 59 articles identified with quantitative data on patterns of injuries in earthquakes, only ten contained pediatric quantitative data, of which only five had a main objective of reporting pediatric injuries. This challenge is encountered worldwide, even for individual injuries. For example, in the U.S. the National Pediatric Trauma Registry (NPTR), a multi-state pediatric-specific trauma registry was discontinued in 2002 due to the lack of funding.^14^ The Trauma Data Bank remains the largest repository of trauma records in the United States; however, it has not focused specifically on pediatric data collection.

The fifth challenge is the feasibility of comprehensive data registry systems in the aftermath of large scale disasters. In such chaotic situations, it can be very difficult to collect sufficient patient information.

Interesting areas of future research, beyond the scope of this paper, include considering the scale of hazard, e.g. the magnitude of earthquakes and seismic intensity in the sites, and vulnerability, e.g. basic infrastructure, health services, and building codes in the sites. These factors can influence the type and severity of the injuries. In addition, a comparison should be made between the characteristics of the earthquake-related injuries among the paediatric population and the earthquake-related injuries among the general population and with those of the paediatric population in usual settings.

## Conclusions

Differences in age group definitions of pediatric patients, and in the injury classification system contribute to difficulty in quantifying the burden of earthquake-related injuries in the pediatric population. Uniform age limits and injury classification systems are paramount for drawing broader conclusions, enhancing disaster preparation for future earthquake disasters and decreasing morbidity and mortality. Some of these conclusions may be applicable to other types of disasters causing pediatric injuries. Further research in the area of pediatric trauma registries in disaster settings is require.

## Appendix 2

### PRISMA Checklist

Download PDF
